# Anesthesia quality indicators to measure and improve your practice: a modified delphi study

**DOI:** 10.1186/s12871-023-02195-w

**Published:** 2023-07-31

**Authors:** May-Sann Yee, Jordan Tarshis

**Affiliations:** 1 Southlake Regional Health Centre, Newmarket, ON L3Y 2P9 Canada; 2 Sunnybrook Health Sciences Centre, University of Toronto, Toronto, ON Canada

**Keywords:** Anesthesia quality indicators, Continuing medical education, Practice improvement, Anesthesia information management system

## Abstract

**Background:**

Implementation of the new competency-based post-graduate medical education curriculum has renewed the push by medical regulatory bodies in Canada to strongly advocate and/or mandate continuous quality improvement (cQI) for all physicians. Electronic anesthesia information management systems contain vast amounts of information yet it is unclear how this information could be used to promote cQI for practicing anesthesiologists. The aim of this study was to create a refined list of meaningful anesthesia quality indicators to assist anesthesiologists in the process of continuous self-assessment and feedback of their practice.

**Methods:**

An initial list of quality indicators was created though a literature search. A modified-Delphi (mDelphi) method was used to rank these indicators and achieve consensus on those indicators considered to be most relevant. Fourteen anesthesiologists representing different regions across Canada participated in the panel.

**Results:**

The initial list contained 132 items and through 3 rounds of mDelphi the panelists selected 56 items from the list that they believed to be top priority. In the fourth round, a subset of 20 of these indicators were ranked as highest priority. The list included items related to process, structure and outcome.

**Conclusion:**

This ranked list of anesthesia quality indicators from this modified Delphi study could aid clinicians in their individual practice assessments for continuous quality improvement mandated by Canadian medical regulatory bodies. Feasibility and usability of these quality indicators, and the significance of process versus outcome measures in assessment, are areas of future research.

**Supplementary Information:**

The online version contains supplementary material available at 10.1186/s12871-023-02195-w.

## Background

Continuing professional development (CPD) refers to the ongoing process of developing new knowledge, skills, and competencies necessary to maintain and improve professional practice. Continuing quality improvement (cQI) is a systematic approach to assessing and improving quality of care by professionals which involves collecting data on indicators of quality and using this data to identify areas for improvement and develop strategies to address them [1]. Anesthesia quality indicators are specific measures used to assess clinical care in anesthesia. Ongoing learning and professional development with change implementation informed by regular feedback using quality indicators that are transparent, reliable, evidence-based, measurable, and improvable is critical to ensuring anesthesiologists continue to provide high-quality and relevant care that meets the needs of their patients.

Recent changes in post-graduate medical education (PGME) training in Canada have necessitated changes in continuing professional development (CPD) requirements for practicing clinicians. While the adoption of competency-based education has fully penetrated anesthesia postgraduate medical education (PGME) training programs in Canada, it is in much earlier stages of implementation in the continuing education realm beyond PGME. The current Royal College of Physicians and Surgeons of Canada (RCPSC) Maintenance of Certification (MOC) program, the national CPD program for specialists, states that, “All licensed physicians in Canada must participate in a recognized revalidation process in which they demonstrate their commitment to continued competent performance in a framework that is fair, relevant, inclusive, transferable, and formative” [1]. This mandate for continuing quality improvement (cQI) applies not only to physicians but also to the national specialty societies providing continuing professional development resources to their physician members.

The Federation of Medical Regulatory Authorities of Canada (FMRAC) published a document titled, “Physician Practice Improvement” in 2016, with the goal of supporting physicians in their continuous commitment to improve their practice [2]. Their suggested five-step iterative process involves (1) understanding your practice, (2) assessing your practice, (3) creating a learning plan, (4) implementing the learning plan, and (5) evaluating the outcomes.

In 2018, the CPD report from The Future of Medical Education in Canada (FMEC) project was published [3]. In this report, principle #2 states, “The new continuing professional development (CPD) system must be informed by scientific evidence and practice-based data” and should, “…encourage practitioners to look outward, harness the value of external data, and focus on how these data should be received and used”, stressing the importance of the data being from physicians’ own practices.

Although these reports make clear a link between competency-based continuing professional development as a physician in practice and the importance of gathering and analyzing physician specific data, it neither provides guidance on what data is relevant for anesthesia, nor how to gather it. While many national organizations including the Canadian Anesthesiologists’ Society publish Guidelines for the Practice of Anesthesia [4], these guidelines are distinct from practice quality indicators. Internationally, national anesthesia specialty societies and safety groups have published lists of anesthesia quality indicators, but the evidence for many of these indicators is weak and not broad-based. Haller et al. published a systematic review of quality indicators in anesthesia in 2009; however, the focus was neither on physician CPD nor cQI [5]. An important distinction exists between the goal of this study and from that of the recent Standardized Endpoints in Perioperative Medicine and the Core Outcome Measures in Perioperative and Anesthetic Care (StEP-COMPAC) initiative, which focused on establishing clear definitions for outcomes for clinical trials [6–10], and not for physician performance improvement.

Therefore, a need currently exists for a list of quality indicators that are relevant to physicians’ goals of continuing quality improvement and ongoing professional development. Furthermore, as electronic anesthesia information management systems (AIMS) become ubiquitous, it is essential that a list of indicators relevant to individuals and the anesthesia community be developed to forward the goal of competency-based CPD. Ideally these indicators would be readily extractable from an AIMS. The purpose of this study was to create a list of anesthesia quality indicators for anesthesiologists to help guide self-assessment and continuing quality improvement.

## Methods

This study received Johns Hopkins Institutional Review Board application acknowledgement (HIRB00008519) on May 27, 2019.

The original Delphi method, first described by Dakley and Helmer in 1962 [11], was used as a method to generate specific information for United States National Defense using a panel of selected experts starting with an open questionnaire. The modified Delphi technique was used to streamline the time and effort of the participants, and the modification involved starting with a pre-selected set of items identified by a literature search rather than with an open questionnaire.

The literature search was performed with the help of a medical health informationist by a review of the literature published between 2009 and 2019 in Pubmed, including Ovid Medline and Cochrane content, using the search protocol outlined in Supplementary Table S1.

Retrieved articles were reviewed by the principal author to determine the relevance to the topic. Inclusion criteria included items deemed to be anesthesia quality indicators in systematic reviews completed within 10 years of the study start date, anesthesia quality indicators currently in use in Canadian academic institutions, anesthesia quality and safety indicators in published articles in peer-reviewed journals, anesthesia quality indicators identified in the Anesthesia Quality Institute National Anesthesia Clinical Outcomes Registry, as well as any additional items generated by the panel. The list of anesthesia quality indicators was reviewed by the second author prior to distribution. The focus on the last ten years of published data helped ensure that the indicators were the most up-to-date available.

Selection of the Delphi panel was based on a stratified random sampling technique [11]. Anesthesiologists representing the different regions across Canada were identified and approached based on their active involvement in the Canadian Anesthesiologists’ Society Continuing Education & Professional Development Committee, Quality & Patient Safety Committee, Standards Committee, Association of Canadian University Departments of Anesthesia Education Committee, the Royal College of Physicians and Surgeons of Canada Specialty Committee in Anesthesiology, or academic involvement. A minimum of 12 participants was sought to ensure validity of the responses [12]. Written informed consent was obtained from all participants.

The survey was created using a matrix table question type with a 2-point binary scale (agree/disagree) with a single answer option. The survey was optimized for mobile devices and each item had an adjacent textbox for comments. Additional items and general comments were solicited at the end of each survey round. A reiteration of the study purpose, research questions, and instructions were emailed to participants along with an anonymous link to the survey. The surveys required 10 to 15 min to complete. Panelists were given a window of 2 weeks to complete each survey round, with 4 weeks between each round.

All responses were gathered anonymously and tallied by Qualtrics survey collection and analysis software (Johns Hopkins University access). Consensus was *a priori* defined as agreement of greater than 70% of the group. With 14 panelists, a consensus was equivalent to 10 or more points on any given item. Subsequent Delphi rounds were planned to continue until stability with less than 15% change in responses from the previous round was achieved. Items that reached consensus would be removed and not recirculated. New items generated by the panelists, items that did not reach consensus, and panelist comments were shared anonymously in subsequent rounds.

## Results

A total of 28 articles published on anesthesia quality indicators from 2009 to 2019 were identified. A subset of these articles was useful for item generation [5–10, 13–30], including several systematic reviews [5, 6, 8–10, 21, 25, 30]. Review of the American Society of Anesthesiologists Anesthesia Quality Institute and Wake Up Safe websites, as well as communication with anesthesia quality experts (separate from the study panel) from two academic centers, provided additional information. A total of 132 anesthesia quality indicators were identified for the initial round of the study. These indicators are presented in Supplementary Table S2.

Twenty-one Canadian anesthesiologists were approached and fourteen consented to participate. A consent form was emailed to those who expressed interest in participating and those who returned a signed consent form were included in the study.

An expert is a person who has a high degree of skill and knowledge in a particular field or subject, acquired through training, education, and experience. They are considered to be authoritative and capable of providing valuable advice and guidance in their area or expertise. The members of the expert panel for this study were identified based on their ongoing involvement with the Canadian Anesthesiologists’ Society (CAS) Continuing Education and Professional Development Committee, the CAS Quality & Patient Safety Committee, the CAS Standards Committee, the Canadian Journal of Anesthesia editorial board, the Royal College of Physicians & Surgeons of Canada Specialty Committee in Anesthesiology, the Association of Canadian University Departments of Anesthesia Education Committee, and University of Toronto Department of Anesthesiology & Pain Medicine faculty.

This expert panel was representative of the different regions across Canada (British Columbia 1; Alberta 2; Manitoba 3; Ontario 4; Quebec 1; Nova Scotia 1; Newfoundland 2). The group spanned all levels of practice with 2 members in practice for < 5 years, 2 members between 5 and 10 years in practice, and the remaining 10 members in practice for > 10 years. There were 7 self-identified females and 7 self-identified males on the panel (Table 1. Panelist Demographics).

**Table 1:** Panelist Demographics. Abbreviations: CAS – Canadian Anesthesiologists’ Society, CEPD – Continuing Education & Professional Development, UBC – University of British Columbia, ACUDA – Association of Canadian University Departments of Anesthesia QPS - Quality and Patient Safety, CJA – Canadian Journal of Anesthesia, CPD – Continuing Professional Development

Panelist ID	Geographic Location	Gender	Committee	Years in Practice
1	British Columbia	Male	CAS CEPD UBC	> 10
2	Alberta	Male	University of Alberta	> 10
3	Alberta	Male	University of Alberta	> 10
4	Manitoba	Male	ACUDA CPD	> 10
5	Manitoba	Female	CAS QPS	< 5
6	Manitoba	Male	CJA	> 10
7	Ontario	Female	University of Ottawa	> 10
8	Ontario	Male	CJA	> 10
9	Ontario	Female	University of Toronto	< 5
10	Ontario	Female	Royal College	> 10
11	Quebec	Female	CAS CEPD	> 10
12	Nova Scotia	Female	CAS Standards Committee	5–10
13	Newfoundland	Male	ACUDA CPD	> 10
14	Newfoundland	Female	ACUDA CPD	5–10

QPS – Quality and Patient Safety, CJA – Canadian Journal of Anesthesia, CPD – Continuing Professional Development

### Iterations

For Rounds 1 through 3, expert panelists were given the following instructions, “*The following items are elements of quality in anesthesia care. Please evaluate each item or event to determine if you think it is reasonable and appropriate for use as a measure of an individual anesthesiologist’s practice by ticking ‘agree’ or ‘disagree*”.

#### Round 1

One hundred thirty-two indicators were circulated to the panel in the initial round. Thirteen out of 14 participants (93%) responded to the survey. Item response rate variability; one hundred twenty-one items had 13 respondents, 10 items had 12 respondents, and 1 item had 11 respondents. Consensus (> 70%) was achieved for 85 items (83 accept; 2 reject). The item with only 11 responses reached consensus to reject. The 85 items that reached consensus were removed from the list and 47 items remained. By combining the 47 remaining items with 9 new items generated from the panel, a total of 56 items were prepared for circulation in the next round.

#### Round 2

Fifty-six items were circulated. Twelve out of 14 participants (86%) responded. Item response rate variability: 50 items had 12 respondents; 6 items had 11 respondents. Consensus (> 70%) was achieved for 13 items (11 accept; 2 reject). The 13 items that reached consensus were removed from the list and 43 items remained. Since the process produced duplicate items in concept, with differing wording, both authors reviewed and curated the list to combine duplicate items without adding or removing any concepts, and in so doing the list was condensed down to 37 items. An example of this process is that failed spinal block, incomplete spinal block, and postdural puncture headache were 3 separate items and were combined into a single item, “complications of neuraxial block”.

#### Round 3

Of the 37 items circulated, consensus was achieved for 7 items (5 accept; 2 reject). Thirteen out of 14 participants (93%) responded. Item response rate variability: 35 items had 13 respondents; 2 items had 12 respondents. The thirty items that did not reach consensus were eliminated.

**Fig. 1:**
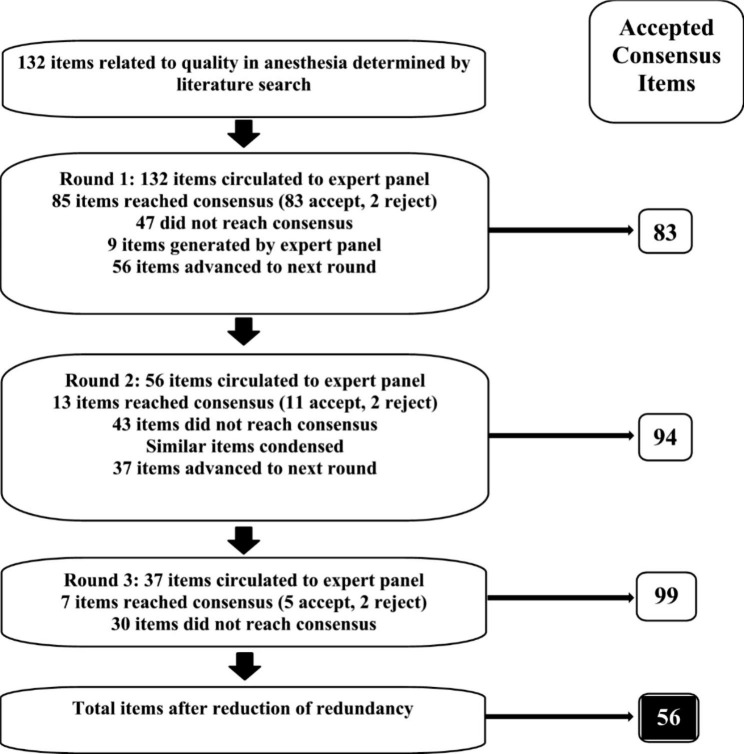
Modified Delphi results summary

After 3 rounds, a total of 132 items were evaluated. Ninety-nine items were accepted with greater than 70% consensus. Six items out of 132 were rejected with greater than 70% consensus. Nine new items were generated from the panel. Items that reached consensus were not recirculated to panelists. Significant redundancy in the 99 items that reached consensus was eliminated by combining items, reducing the list to 56 items (Fig. 1).

There was a 10-month pause between rounds 3 and 4 due to Covid19 pandemic related disruptions. In the 4th round, the 56-item list was sent out to the study panel with specific instructions to “*select 20 anesthesia quality indicators from the list below that you believe to be of top priority in the continuous self-assessment and feedback of an anesthesiologists’ practice*”. The electronic survey tool required exactly 20 responses, ranking of these 20 responses was not required. All 14 study panelists responded in the Round 4. Table 2 ranks the 56 indicators according to the number of votes received from the panel in Round 4.

**Table 2:** List of anaesthesia quality indicators ranked by response in the fourth round of the mDelphi. The % column is the percent of respondents who ranked the indicator in the top 20. Similar items are grouped together though deemed sufficiently different to list separately. An asterisk marks the items deemed most easily extractable from and EMR/AIMS

Indicator	n/14	%	Donabedian Quality Classification
Airway complications (greater than 3 attempts at intubation, cannot intubate/cannot ventilate, laryngospasm, hypoxia, dental/soft tissue injury)∗	13	93	Process & Outcome
Incidence & duration of perioperative adverse events including hypoxia, hyper/hypocarbia, hyper/hypothermia, hyper/hypoglycemia, anesthetic overdose∗ Degree & duration of hypotension on induction (SBP < 80) ∗	12 10	86 71	Outcome Outcome
Number of medical errors (patient receiving wrong drug, drug dose, wrong surgical site, wrong blood product etc) Medication error with wrong medication or wrong dose given Number of wrong site or side procedures	11 10 1	79 71 < 1	Process Process Process/Structure?
Patient satisfaction (composite patient experience)	11	79	Outcome
Postoperative residual neuromuscular blockade (ToF < 0.9 measured 15 min after arrival to PACU, clinical residual weakness) requiring intervention by an anesthesiologist to treat inadequate reversal of neuromuscular blockade∗	10	71	Outcome
Temperature less than 35.5 Celsius on arrival to PACU∗	10	71	Outcome
Complications of neuraxial block (failed block, inadvertent dural puncture, high block, infection, neurologic complication etc) ∗ Inadequate regional/neuraxial block requiring supplemental analgesia, sedation or conversion to GA required for surgery∗ Epidural or neuraxial technique not working as planned, inadequate pain coverage necessitating additional interventions	9 6 2	64 43 14	Outcome Outcome/Process Outcome
Incidence of severe postoperative nausea and vomiting (2 or more episodes of severe nausea/vomiting over 6 hours apart OR requiring more than 2 doses of antiemetics; patients who receive an intervention by an anesthesiologist for PONV not responding to PACU protocols in the recovery period) ∗	9	64	Outcome
Complications of central venous line placement (arterial puncture, pneumothorax, nerve injury, infection, etc) ∗	8	57	Outcome
Intervention required by an anesthesiologist in PACU to relieve respiratory distress∗	8	57	Outcome & Process
Perioperative cardiac complications (arrhythmia, ischemia, myocardial infarction) ∗	7	50	Outcome
Adequacy of postoperative pain management (pain scores at defined intervals, plan documentation, care pathway milestones, etc) ∗	7	50	Outcome
All cause complications within 30 days or until hospital discharge or death	7	50	Outcome
Proportion of patients receiving appropriate surgical antibiotic prophylaxis∗	7	50	Process & Structure
Unplanned overnight admission of day surgery patients for anesthetic reasons	7	50	Outcome
Pain scores on arrival to PACU∗	7	50	Outcome
Cerebrovascular accident developing during or within 48h of anesthetic care	6	43	Outcome
Monitoring hand hygiene (direct observation or mechanical monitoring)	6	43	Process & Structure
Adequate perioperative management of patient’s current medications	6	43	Process
Incidence of intraoperative awareness	6	43	Outcome
Incidence of delirium during the postoperative period	6	43	Outcome
Patient requiring an intervention by an anesthesiologist for circulatory/hemodynamic reasons in the recovery period∗	5	36	Outcome
Critical incident reviews for rare and infrequently occurring events	5	36	Process & Structure
Ultrasound used for vascular access procedures∗	5	36	Process
Unplanned admission to ICU or high dependency unit within 24h of a procedure involving anesthesiology	5	36	Outcome
Proportion of charts with documentation of informed consent and risks on anesthetic technique∗	4	29	Process & Structure
Patients developing severe respiratory depression requiring naloxone administration during acute pain management∗	4	29	Outcome & Process
Unplanned extended PACU stay for medical reasons	4	29	Outcome
Adherence to care pathway processes (eg. ERAS or fast track protocols)	4	29	Process
Percentage of cases receiving PONV prophylaxis∗	4	29	Process
Renal insufficiency (25% increase in serum creatinine or absolute increase > 44umol/L at any time within the first 5 postop days) or renal failure (doubling of serum creatinine or oliguria < 500ml/24h developing during or within 48h or anesthetic care)	4	29	Outcome
Visual loss or eye injury∗	3	21	Outcome
360 degree evaluation	3	21	Structure & Process
ASA physical status class∗	3	21	N/A
Time to patient orientation (able to correctly answer name and whereabouts)	3	21	Outcome
Surgical safety checklist completed before induction∗	2	14	Process & Structure
Preoperative patient anxiety adequately addressed by anesthesiologist (eg. Bauer questionnaire 24h postop)	2	14	Outcome & Process
Caseload breakdown by anesthetic technique (GA, neuraxial, regional, sedation, local, none, etc) ∗	2	14	Process
Adverse drug reaction other than anaphylaxis∗	2	14	Outcome
Transfusion-related complications (volume overload, TRALI, ABO incompatibility)	2	14	Outcome
Noncardiogenic pulmonary edema during or within 48h or anesthetic care	2	14	Outcome
Surgical service∗	2	14	N/A
Surgical priority (emergent, urgent, elective) ∗	2	14	N/A
Surgical caseload (time of day, day of week) ∗	2	14	N/A
Number of GA cases using neuromuscular blockade where reversal was given intraoperatively∗	2	14	Process
Epidural or neuraxial technique not working as planned, inadequate pain coverage necessitating additional interventions	2	14	Outcome
General anesthetic given for a Cesarean section∗	1	< 1	Process
Proportion of patient with clearly documented transfer of care immediately postoperatively to a PACU or ICU (eg. checklist) ∗	1	< 1	Process
Incidence of local anesthetic toxicity∗	0	0	Outcome
Number of patients receiving a blood transfusion in OR or PACU∗	0	0	Outcome
Number of cases where PACU was bypassed∗	0	0	Process & Structure

Abbreviations: ToF train-of-four; PACU post-anesthesia recovery unit; SBP systolic blood pressure; PONV postoperative nausea & vomiting; GA general anesthesia; ICU intensive care unit; ERAS early recovery after surgery; TRALI transfusion-related acute lung injury; OR operating room; N/A not applicable

## Discussion

The overall goal of this initiative was to answer the question of whether in the current era of competency-based medical education and the increasing use of electronic medical records and AIMS, can a consensus list of indicators be identified to aid clinicians and Departments in promoting practice and performance improvement by measuring, analyzing, and using the data to improve the quality of anesthetic care. This process requires establishing a list of anesthesia quality indicators as an essential first step. Our study determined that airway complications, incidence & duration of perioperative adverse events, number of medical errors, patient satisfaction, perioperative residual neuromuscular blockade requiring intervention by an anesthesiologist, patient temperature less than 35.5 Celsius on arrival to PACU, complications of or failed neuraxial block, and incidence of severe PONV to be the most important anesthesia specific quality indicators for continuous self-assessment and feedback of an anesthesiologist’s practice.

It is useful to determine the type of categories under which these quality indicators can be grouped. In a seminal manuscript, Donabedian categorized quality indicators into 3 groups: structure (supportive and administrative), process (provision of care), and outcomes (measurable and patient related) [31]. In their 2009 systematic review Haller et al. identified 108 quality indicators (only 40% of which were validated beyond face validity), and found that 57% were outcome metrics, 42% measured process of care metrics, and 1% were structure-related metrics [5]. Hamilton et al. (2021) reviewed regional anesthesia quality indicators and found that 76% of 68 identified items were outcome measures, 18% process of care measures, and 6% structure-related [25]. Our findings identified 56 consensus quality indicators, 52% were outcome-related, 35% were process-related, and 12% were structure-related indicators. This is in agreement with other studies, with the top results being primarily outcome indicators, followed by process and then structure. Process indicators in anesthesia can be difficult to measure because there is variability in practice between providers and healthcare settings that make it difficult to develop standardized processes. Anesthesia care is a complex process with multiple steps making measuring and tracking time-consuming and resource intensive. Smaller health care settings and outpatient procedures may have limited opportunity to collect data on anesthesia processes. There is also a lack of consensus among healthcare providers regarding the most important process to measure and track in anesthesia care. For all these reasons, there are relatively less process indicators compared to outcome quality indicators in anesthesia.

Demographic indicators were included in the outset of this study because items such as surgical service, surgical priority, ASA status, caseload, number of GA cases, number of spinals provide a snapshot of an individual anesthesiologist’s practice and serves to help clinicians understand and assess their practice by following the first 2 steps of the FMRAC’s 5-step iterative process to practice improvement: (1) understanding your practice and (2) assessing your practice.

Perioperative mortality is a notably absent quality indicator in this study. Benn et al. noted that as the anesthesia specialty has been at the forefront of improving safety in healthcare, significant morbidity and mortality attributable to anesthesia has decreased significantly over the last half century. Mortality is a poor anesthesia quality indicator because it is rare and usually related to factors outside the anesthesiologists’ control. Data from the UK reveals that less than 1% of all patients undergoing surgery die during the same hospital admission and perioperative mortality of a healthy elective patient undergoing surgery is a mere 0.2% [32].

Relying on expert opinion and consensus, the modified Delphi technique was intentionally chosen for this study because a strong level of evidence for most anesthesia quality indicators is lacking. Expert opinion, therefore, provides a level of face validity. Advantages of the modification, include improved initial round response rates, solid grounding in previously developed work, reduced effect of bias due to group interaction, and assured anonymity while providing controlled feedback to participants [33]. The variable item response rates on the Delphi rounds are a common challenge to this method despite measures to prevent panel attrition including, (1) ensuring each round required less than 15 min to complete, (2) not recirculating items that reached consensus, and (3) using two options agree/disagree rather than a rank scale (e.g. Likert). Large datasets containing many items is a recognized challenge. However, previous attempts to reduce fatigue by creating competency subsets, sub-panels, or rotational modifications were largely unsuccessful, resulting in an increased number of rounds and introduction of bias, while being subject to the same factors that threaten the validity of any Delphi study (lack of experts on the panel, lack of clear content definition, poorly developed initial dataset) [34]. The item response rates of the 14-member panel ranged between 86 and 100% indicating that there was a consistent level of interest among the group members in participating in this study.

The modified Delphi study begins with a list of pre-selected items, but also gives panel members opportunity to generate new items. The elements of quality can be used to define what is considered to be good quality, and the specific quality indicators can be selected to measure and track each element. The term ‘element of quality’ was used in the instruction to panel members to keep the process open, inclusive, and as broad-based as possible as there may be newly emerging elements of anesthesia care or that have yet to be fully defined or properly studied, that could be added to the list for consideration.

Using quality indicators with the intent of providing effective feedback to improve quality requires that indicators be transparent, reliable, evidence-based, measurable, and improvable. Feedback processes should be regular, continuously updated, comparative to peers, non-judgmental, confidential, and from a credible source [32]. EMR/AIMS is an excellent source of data with these qualities yet requires time, technological skills, and institutional financial investments to initiate and maintain. The intent of this study was to focus on quality indicators extractable from an EMR/AIMS, and the participants were informed of this goal in the introduction to this study. Nonetheless, many of the indicators proposed by the participants are broad and may not be easily extractable from an electronic system. The use of EMR/AIMS in Canada at the time of the study was highly variable in both the availability of use and the specific software being used and may have contributed to the generated item list not including exclusively extractable items. This discrepancy between intent and outcomes of this study are indicative of the challenges of identifying, gathering, and distilling the massive quantity of extractable data from an EMR/AIMS.

There were several limitations to this study. The final list of generated items in Table 1 has been reviewed and items marked with an asterisk have been deemed to be most likely to be extractable from an EMR, based on the quantitative nature of the item, recognizing there is heterogeneity in the data mining capabilities of various electronic records and that the quality of data extraction is directly related to the quality and detail of input data. For example, aspects of care which are multi-dimensional, such as patient satisfaction would be more difficult to extract from most EMR’s compared to a concise, focused element, such as measured temperature of less than 35.5 Celsius on arrival in the post anesthetic care unit. Some institutions might consider an automated dashboard using indicators which would require efforts to set up but once in place could provide ongoing, on-demand clinician feedback [35]. Quality indicators can be used in a balanced score card or a quality clinical dashboard for the purposes of continuing quality improvement. The balanced scorecard approach functions by linking clinical indicators to an organization’s mission and strategy in a multi-dimensional framework. A quality clinical dashboard is used to provide clinicians with relevant and timely information that informs decisions and helps monitor and improve patient care [26]. Regardless of the feedback methods, effecting lasting change in clinician practice and patient outcomes can be challenging.

Although efforts were made to obtain broad national geographic representation of participants and individuals were chosen based on their background in education and quality improvement, it is recognized that some valuable data may have been overlooked by not including allied health workers and patients in this study. However, continuous performance improvement, a form of Lean Improvement [36], emphasizes the tenets that ideas for improvement originate from people who do the work and that it is essential to understand the work process before trying to fix it. Additionally, a recent study by Bamber et al. [27]., found that both allied health members and patients included in their study demonstrated significant participant attrition of both these groups through the Delphi process. A consensus face-to-face meeting was not included in our study to reduce the risk of nuances lost in virtual meetings during the pandemic and because the panel had anesthesiologists of different career stages, to mitigate the potential influence of senior panelists on junior panelists voicing differing opinions and to reduce the risk of ‘group think’.

Our study was paused after the third round to avoid attrition as the participants were dealing the clinical challenges at the onset of the COVID-19 pandemic. The fourth and last round of this study is a slight deviation from the original study methods and was decided on after the authors recognized the need to prioritize the list of indicators. Twenty items were chosen to aid the reader in prioritizing these indicators.

While there remain questions regarding how these indicators can be best used, as well as hurdles related to cost of implementation and end-user buy-in, it is recognized that comprehensive practice assessment must be based on more than data collected from an electronic record. The next steps in this project would be to further refine those indicators that are both feasible to collect and most desirable to end users.

## Conclusion

This study has identified and prioritized a list of 56 anesthesia quality indicators deemed to be both relevant to an anesthesiologist’s practice and obtainable from an electronic record. This is an essential step in the goal of aiding clinicians and departments in meeting ongoing cQI requirements recommended by professional societies and medical regulatory bodies.

## Electronic supplementary material

Below is the link to the electronic supplementary material.

Supplementary Material 1

Supplementary Material 2

## Data Availability

The datasets generated and/or analyzed during the current study are not publicly available because the institutional rules strictly prohibit releasing the native data on the web but are available from the corresponding author on reasonable request.

## Abbreviations

CPD: continuing professional development
MOC: maintenance of certification
RCPSC: Royal College of Physicians and Surgeons of Canada
AQI: anesthesia quality indicators
EMR: electronic medical records
AIMS: anesthesia information management system
mDelphi: modified Delphi
ASA: American Society of Anesthesia
cQI: continuing quality improvement
FMRAC: Federation of Medical Regulatory Authorities of Canada
GA: general anesthesia
PGME: post-graduate medical education
