# Supplementary material for: Anesthesia quality indicators to measure and improve your practice: a modified delphi study
Source: BMC Anesthesiol. 2023 Jul 31;23:256. doi: 10.1186/s12871-023-02195-w (PMC10388503; doi:10.1186/s12871-023-02195-w)
Supplement: Supplementary file 1 — Supplementary Material 1 [file 12871_2023_2195_MOESM1_ESM.docx]

**Supplementary Table S1**. Literature search strategy

| Search Parameters | Search Results |
| --- | --- |
| [Mesh] “Anaesthesia” AND Guideline, last 10 years | 193 |
| [Mesh] “Anaesthesia” AND Guideline, last 10 years, English | 148 |
| [Mesh] “Anaesthesia” AND Guideline AND indicat*, last 10 years | 16 |
| [Mesh] “Anaesthesia” AND Indicator*[TI], last 10 years, English | 107 |
| [Mesh] “Anaesthesia” AND “quality indicator”, last 10 years, English | 47 |
| [Mesh] “Anaesthesia” AND “safety indicator”, last 10 years, English | 3 |
| [Mesh] “Anaesthesia” AND “quality and safety”, last 10 years, English | 43 |
| [Mesh] “Anaesthesia” AND “quality indicators”, last 10 years, English | 44 |
| [Mesh] “Anaesthesia” AND “safety indicators”, last 10 years, English | 2 |
| [Mesh] “Anaesthesia” AND “standardized endpoints”, last 10 years, English | 0 |
| (operat* OR surg*) AND anesthesia(Mesh) AND standardized endpoint, last 10 years, English | 21 |
| Standardized endpoint AND perioperative AND anesthesia, last 10 years, English | 72 |
