# Supplementary material for: Anesthesia quality indicators to measure and improve your practice: a modified delphi study
Source: BMC Anesthesiol. 2023 Jul 31;23:256. doi: 10.1186/s12871-023-02195-w (PMC10388503; doi:10.1186/s12871-023-02195-w)
Supplement: Supplementary file 2 — Supplementary Material 2 [file 12871_2023_2195_MOESM2_ESM.docx]

**Supplementary Table S2**. Original compilation of anesthesia quality indicators used in this mDelphi study

| Surgical priority (eg. Emergent, urgent, elective) | ASA physical status class |
| --- | --- |
| Surgical service | Caseload - time of day, day of week |
| Perioperative antibiotics ordered and given within 60 minutes of incision (2h if fluoroquinolone or vancomycin) | Pain scores on arrival to PACU |
| Prophylaxis for PONV | Number of arterial lines places |
| Number of central lines placed | Frequency of use of alternate airway devices (eg. bougie, Glidescope, FOB) |
| Number of spinal cases | Number of GA cases |
| Postoperative residual NMB (TOF<0.9 measured 15 min after arrival to PACU with presence of muscle weakness, respiratory depression/failure, laryngospasm, or need for reintubation) | Documented pre-anesthesia assessment of the surgical patient before the day of surgery by an anesthesiologist |
| Documented pre-anesthesia assessment of the surgical patient before the day of surgery by the anesthesiologist performing the anesthesia | Documented pre-anesthesia assessment of a patient before surgery by an anesthesiologist for which adequate time has been allowed |
| Prevention of central venous catheter-related blood stream infections (all elements of sterile barrier technique followed) | Consent for the administration of anesthesia or sedation, information on risks documented in the patient chart |
| Unanticipated difficult airway | Written, verbal, or visual information on the anesthesia technique documented in the patient chart |
| Documented evidence of informed consent for labour ward epidural-spinal analgesia | Preoperative EKG obtained according to departmental or other established protocols |
| Patients scheduled for day surgery whose procedure is cancelled on the day of surgery for anesthetic reasons other than acute medical condition | Intraoperative cardiac dysrhythmia or arrest |
| Patient receiving a blood transfusion in accordance with guidelines during the procedure with an anesthesiologist in attendance | Patient who undergoes a procedure with an anesthesiologist in attendance where there is an assistant to the anesthesiologist |
| Adequate preoperative management of patient’s current medications | General anesthetic given for Cesarean section |
| Critical phase distractions minimized by anesthesiologist (ie. “sterile cockpit” concept) | Unplanned overnight admission of day surgery patients for anesthetic reasons |
| Patients who have an unplanned extension to the time between entry into the PACU to the meeting of hospital/day surgery discharge criteria | Post-anesthetic transfer of care: use of checklist of protocol for direct transfer of care from procedure room to intensive care. Unit |
| Incidence of post-dural puncture headache | Patients with analgesia adequate enough to allow acute rehabilitation (eg. Effective cough, mobilization) |
| Assessment of acute post-operative pain | Patients developing severe respiratory depression requiring naloxone administration during acute pain management |
| Post-anesthetic transfer of care measure: procedure room to a post-anesthesia care unit | Anesthesiology counselling about smoking abstinence |
| Neurologic deficit 3 months after procedure secondary to either neuraxial technique or plexus block | Patients developing epidural abscess or epidural hematoma after neuraxial blockade |
| Intervention by an anesthesiologist to relieve respiratory distress in PACU | Intervention by an anesthesiologist to create inadequate reversal or NMB in the recovery period |
| Patient requiring an intervention by an anesthesiologist for circulatory reasons in the recovery period | Incidence of severe PONV (defined as 2 or more episodes of severe nausea/vomiting over 6 hour apart OR requiring >2 ones of antiemetics); patient who receive an intervention by an anesthesiologist for PONV not responding to PACU protocols in the recovery period |
| Patient temperature less than 35.5 Celsius on arrival to PACU | Intervention by an anesthesiologist to manage severe pain not responding to PACU protocol in the recovery period |
| Patient undergoing a procedure with an anesthesiologist in attendance who have an unplanned PACU stay longer than 2h | Death within 48h of a procedure involving anesthesia |
| Operation cancelled while patient receiving anesthetic care | Failed tracheal intubation and inability to ventilate with mask |
| Cardiac arrest (not part of the surgical procedure) during or within 48h of anesthetic care | Acute myocardial infarction during or within 48h of anesthetic care |
| Noncardiogenic pulmonary edema (not part of a medical plan) during or within 48h of anesthetic care | Perioperative aspiration of gastric contents (defined as tracheal pH<4.0 lasting 15s or more, clinical signs of aspiration including coughing/wheezing, decrease in SpO2>10% of baseline preoperative value on room air, radiographic abnormalities within 2h of aspiration) |
| Renal insufficiency (25% increase in serum creatinine or absolute increase >44umol/L at any time within the first 5 post days) or renal failure (doubling of serum creatinine or oliguria <500ml/24h) developing during or within 48h of anesthetic care | Cerebrovascular accident developing during or within 48h of anesthetic care |
| Inadequate regional block (supplemental analgesia, sedation or both required for surgery) | Medication error with the wrong medication given |
| Medication error with the wrong dose being given | Adverse drug reaction other than anaphylaxis |
| Anaphylaxis | Hemolytic blood transfusion reaction resulting from ABO incompatibility |
| Comprehensive planning for pain management documented | Noncardiac surgical patient who received prophylactic antibiotics and who have an order for discontinuation within 48h of surgical end time |
| Composite patient experience | Surgical safety checklist - applicable safety checks completed before induction of anesthesia |
| Prophylactic antibiotic selection for surgical patients according to current recommendations | Cardiac surgery patient with controlled 6am postoperative serum glucose |
| Surgery patients on a beta-blocker before arrival who received a beta-blocker during the preoperative period | Patient with isolated coronary artery bypass graft documented to have received perioperative beta-blockade |
| Intraoperative or postoperative acute myocardial infarction diagnosed during index hospitalization and within 30 days of surgery | Intraoperative or postoperative pulmonary embolism diagnosed during index hospitalization and within 30 days of surgery. |
| Surgery patients who received appropriate venous thromboembolism prophylaxis within 24h before surgery to 24h after surgery | Number of malignant hyperthermia susceptible cases |
| Postoperative pulmonary embolism or deep vein thrombosis | Infection following peripheral nerve block |
| Iatrogenic pneumothorax | Major systemic local anesthetic toxicity |
| After 48h, percentage of patients who rate pain greater than 4 or at an acceptable level to patient | Monitoring of hand hygiene (mechanical monitoring v. direct observation) |
| Number of procedures on the wrong patient or wrong site | Number of patient deaths of major permanent loss of function associated with a problem with medical equipment |
| Number of patient deaths, paralysis, coma, or other major permanent loss of function associated with a medical error | Total perioperative mortality for all ASA physical status classes |
| Complications after sedation and analgesia in intensive care units, cardiac Cath labs, radiology-endoscopy suites, and emergency departments | Perioperative death within 30 days of anesthetic for each ASA class |
| Death within 30 days of a heart bypass operation (in-hospital and after discharge) | Peripheral neurologic deficit following regional anaesthesia |
| Degree and duration of severe hypotension (SBP<80mmHg) on anesthetic induction | Time to fulfillment of criteria for leaving OR (stable vital signs adequate ventilation, following simple commands) |
| Sum of all prespecified complications (renal failure, pulmonary oedema or circulatory failure not related to infection, acute myocardial infarction, stroke, arrhythmia not present perioperatively, cardiac failure, reoperation for suture failure/hemorrhage/evisceration, paralytic ileus, infection) | Surgical site infection within 30 days of surgery |
| Airway (soft tissue or dental) injury as a result of airway manipulation including mucosal trauma, sore throat (constant pain independent of swallowing), dysphonia (difficulty or pain on speaking), dysphagia (difficulty or pain provoked by swallowing) at 24h post | Complication related to invasive line placement |
| Failed regional anaesthesia technique necessitating conversion to GA | Failed spinal block (sensory anesthesia below T6 dermatome 60 minutes after initiation of block) |
| Failed epidural (unable to insert catheter or no sensory block after injection of local anesthetic) | Unintended epidural vessel penetration (free flow of blood or pink return of local anesthetics, franks aspiration of blood, symptoms of tinnitus, drowsiness, metallic taste, light headedness, slurring of speech, perioral numbness, vertigo) following a test dose |
| Incomplete epidural block (unilateral block, unblocked sacral segments, patchy block) | Very high (>T4) or total spinal block due to unintended intrathecal injections |
| Post-dural puncture headache | Number of intubation attempts |
| Failed attempt at intubation | Intraoperative laryngospasm |
| Hypoxia and duration during mask ventilation or tracheal intubation (SpO2<95%) | Visual loss |
| Intraoperative awareness (ie. postop awareness of intraoperative events as determined by mBrice questionnaire administered on post-op day 1 and at post-op day 30) | Time to orientation (ie. able to correctly answer name and whereabouts correctly) |
| Frequency of use of sugammadex | Surgeon satisfaction with surgical conditions (eg. Adequate exposure during one-lung ventilation, laparoscopic exposure) |
| Incidence of perioperative hypoglycemic events | Incident of delirium during the postoperative period |
| Incidence of postoperative hematologic derangements (eg. platelets <50, WBC less than 2.5 or greater than 30, disseminated intravascular coagulation, platelet fall >50% with PTT>20 and increased d-dimer >500) | Postoperative hepatic failure (AST & ALT >200 or total bilirubin >51umol/L) |
| Non-fatal cardiac arrest | Non-fatal myocardial infarction |
| New myocardial schema | New onset symptomatic atrial fibrillation |
| Cardiovascular death within 12 months of surgery | 30-day mortality after heart surgery or at a later time as a direct consequence of a perioperative complication |
| Adjusted 30-day mortality following carotid endarterectomy | Number of patient who died or experienced complications within 30 days or until hospital discharge (wound dehiscence, infection, peritonitis, anastomotic leak, intra-abdominal abscess, pneumonia, UTI, sepsis, MI, CHG/pulmonary edema, respiratory failure needing intubation and ventilatory support, arrhythmias requiring start of new treatment, severe hypotension, cerebrovascular accident, bleeding, bowel obstruction, ARDS, pulmonary emboli, renal dysfunction or failure needing dialysis, death) |
| Eligibility to bypass PACU or ‘fast-tracking’ (mAldrete score: patient activity, respiration, circulation, consciousness, oxygen saturation) | Unplanned admission to ICU or high-dependency unit within 24h of a procedure with an anesthesiologist in attendance (decreased LOC, respiratory arrest, RR>40 or <8/min, low oxygen saturation, respiratory acidosis, inability to clear secretions, hemodynamic instability, inadequate tissue perfusion, requirement of invasive monitoring, use of inotropes or vasopressors, life-threatening arrhythmias) |
| Readmission to hospital within 30 days of surgery | Length of stay |
| Duration of perioperative adverse events including hypoxia, hyper/hypocarbia, hyper/hypothermia, hyper/hypoglycemia, anesthetic agent overdose | Adherence to ERAS protocols including multimodal PONV prophylaxis intraoperative lidocaine infusion, scheduled post-op NSAID use, post opioid administration protocol for breakthrough pain |
| Preoperative patient anxiety adequately addressed by anesthesiologist (Bauer questionnaire posted 24h) | New corneal injury not diagnosed in the PACU/recovery room after anesthesia care |
| Number of epidural/CSE cases | Number of GA cases using NMB where reversal was given intraoperatively |
